# Boosting of HIV envelope CD4 binding site antibodies with long variable heavy third complementarity determining region in the randomized double blind RV305 HIV-1 vaccine trial

**DOI:** 10.1371/journal.ppat.1006182

**Published:** 2017-02-24

**Authors:** David Easterhoff, M. Anthony Moody, Daniela Fera, Hao Cheng, Margaret Ackerman, Kevin Wiehe, Kevin O. Saunders, Justin Pollara, Nathan Vandergrift, Rob Parks, Jerome Kim, Nelson L. Michael, Robert J. O’Connell, Jean-Louis Excler, Merlin L. Robb, Sandhya Vasan, Supachai Rerks-Ngarm, Jaranit Kaewkungwal, Punnee Pitisuttithum, Sorachai Nitayaphan, Faruk Sinangil, James Tartaglia, Sanjay Phogat, Thomas B. Kepler, S. Munir Alam, Hua-Xin Liao, Guido Ferrari, Michael S. Seaman, David C. Montefiori, Georgia D. Tomaras, Stephen C. Harrison, Barton F. Haynes

**Affiliations:** 1 Duke University, Durham, North Carolina, United States of America; 2 Boston Children’s Hospital, Harvard Medical School, Boston, Massachusetts, United States of America; 3 Dartmouth College, Hanover, New Hampshire, United States of America; 4 US Military HIV Research Program, Walter Reed Army Institute of Research, Silver Spring, Maryland, United States of America; 5 U.S. Army Medical Directorate, AFRIMS, Bangkok, Thailand; 6 The Henry M. Jackson Foundation for the Advancement of Military Medicine, Bethesda, Maryland, United States of America; 7 Thai Ministry of Public Health, Nonthaburi, Thailand; 8 Mahidol University, Bangkok, Thailand; 9 Royal Thai Army Component, AFRIMS, Bangkok, Thailand; 10 GSID, South San Francisco, California, United States of America; 11 Sanofi Pasteur, Swiftwater, Pennsylvania, United States of America; 12 Boston University, Boston, Massachusetts, United States of America; 13 Beth Israel Deaconess Medical Center, Harvard Medical School, Boston, Massachusetts, United States of America; Vaccine Research Center, UNITED STATES

## Abstract

The canary pox vector and gp120 vaccine (ALVAC-HIV and AIDSVAX B/E gp120) in the RV144 HIV-1 vaccine trial conferred an estimated 31% vaccine efficacy. Although the vaccine Env AE.A244 gp120 is antigenic for the unmutated common ancestor of V1V2 broadly neutralizing antibody (bnAbs), no plasma bnAb activity was induced. The RV305 (NCT01435135) HIV-1 clinical trial was a placebo-controlled randomized double-blinded study that assessed the safety and efficacy of vaccine boosting on B cell repertoires. HIV-1-uninfected RV144 vaccine recipients were reimmunized 6–8 years later with AIDSVAX B/E gp120 alone, ALVAC-HIV alone, or a combination of ALVAC-HIV and AIDSVAX B/E gp120 in the RV305 trial. Env-specific post-RV144 and RV305 boost memory B cell V_H_ mutation frequencies increased from 2.9% post-RV144 to 6.7% post-RV305. The vaccine was well tolerated with no adverse events reports. While post-boost plasma did not have bnAb activity, the vaccine boosts expanded a pool of envelope CD4 binding site (bs)-reactive memory B cells with long third heavy chain complementarity determining regions (HCDR3) whose germline precursors and affinity matured B cell clonal lineage members neutralized the HIV-1 CRF01 AE tier 2 (difficult to neutralize) primary isolate, CNE8. Electron microscopy of two of these antibodies bound with near-native gp140 trimers showed that they recognized an open conformation of the Env trimer. Although late boosting of RV144 vaccinees expanded a novel pool of neutralizing B cell clonal lineages, we hypothesize that boosts with stably closed trimers would be necessary to elicit antibodies with greater breadth of tier 2 HIV-1 strains.

**Trial Registration:** ClinicalTrials.gov NCT01435135

## Author summary

Developing a successful HIV-1 vaccine remains a high global health priority. Several HIV-1 vaccine trials have been performed with only the RV144 vaccine trial showing vaccine efficacy, albeit modest. No broadly neutralizing antibody activity was identified in RV144 and inducing sterilizing immunity against a complex pathogen like HIV-1 remains a major challenge. Here we characterize the B cell responses after RV144 vaccine-recipients received two additional boosts severals years after the conclusion of the RV144 vaccine trial. Delayed and repetitive boosting of RV144 vaccine-recipients was capable of increasing somatic hypermutation of the Env-reactive antibodies and expanding subdominant pools of neutralizing B cell clonal lineages. These data are pertinent to HIV-1 vaccine-regimen design.

## Introduction

Six HIV-1 vaccine efficacy trials have been performed [1–5], of which only one, the ALVAC-HIV and AIDSVAX B/E prime-boost RV144 trial, showed vaccine protection, with estimated vaccine efficacies of 60% at 12 months [6] and 31% at 42 months [7]. Plasma IgG antibodies binding to HIV-1 envelope variable region 2 (V2) and low Env IgA binding levels were immune correlates of decreased transmission risk [8]. V2-specific antibodies isolated from RV144 bound tier 2 HIV-1 infected CD4 T cells and mediated antibody dependent cellular cytotoxicity (ADCC) [9].

While no broadly neutralizing antibodies (bnAbs) were induced in RV144 [8,10] the induction of bnAbs remains a prime goal of HIV vaccine development, since passive administration of bnAbs has repeatedly shown to protect against simian HIV-1 (SHIV) chimeric virus challenge [11–15]. BnAbs develop in approximately 50% of HIV-1 infected individuals, but these arise only after several years of infection [16,17]. One hypothesis to explain why HIV-1 bnAbs have been difficult to induce by vaccination is that these antibodies have one or more unusual characteristic—long HCDR3 regions, autoreactivity with host antigens, and/or extensive somatic mutations—all traits of antibodies controlled by host tolerance control mechanisms [18–22]. A result of tolerance control of bnAbs is that bnAb precursors may be reduced in frequency in the pre-vaccination B cell repertoire; they may also be at a competitive disadvantage with other more dominant precursor B cell pools. For these reasons, inducing bnAbs may require an extensive vaccination-regimen.

Here we sought to determine if a pool of subdominant B cells, such as those that produce long HCDR3 CD4 bs bnAbs, may be expanded when an Env immunogen that binds bnAb UCAs is included in a boosting regimen. In the RV305 clinical trial, RV144 vaccine-recipients who had previously received the initial ALVAC-HIV + AIDSVAX B/E gp120 immunization regimen (0,1,3,6 months) and remained HIV-1- uninfected were boosted with ALVAC-HIV, AIDSVAX B/E gp120, or ALVAC-HIV + AIDSVAX B/E gp120 6–8 years later (S1 Fig). We found that boosting of RV144 vaccinees led to an increased frequency of memory B cells producing envelope-specific antibodies with long HCDR3s. Several of the mature antibodies and inferred unmutated common ancestors (UCA) neutralized both neutralization sensitive HIV-1 isolates (tier 1) and a difficult-to-neutralize (tier 2) HIV-1 CRF01 AE isolate, CNE8.

## Results

### AIDSVAX B/E gp120 boosted antibodies with long HCDR3 regions

After two boosts (6-month interval) with the same immunogens 6–8 years after the completion of the RV144 primary immunizations (S1 Fig), plasma neutralizing antibody (nAb) responses were assayed in the A3R5 pseudovirus neutralization assay [23] against a panel of 11 CRF01 AE isolates (S2A Fig). Previous work has shown that neutralization of neutralization resistant (tier 2) HIV-1 isolates by antibodies is more readily detected in the A3R5 cell based assay than in the TZM-bl cell based assay [23]. Here the A3R5 cell based assay was used to search for vaccinees who had robust antibody responses to Env. We selected four vaccinees for study who had high magnitude and breadth of neutralization. Two were from RV305 Group 1 who received ALVAC-HIV plus AIDSVAX B/E gp120 boosts (3043, 3070), and two were from RV305 Group 2 who received only AIDSVAX B/E gp120 boosts (3064, 3053) (S2B Fig). In all four vaccinees, the RV305 boosts increased both autologous (AE.A244gp120) and heterologous (B.6240gp120) plasma IgG-gp120 binding responses, to levels higher than those observed after the initial RV144 regimen (S3A Fig) The RV305 boosts also increased the magnitude of B.MN and AE.92TH023 neutralization in the TZM-bl neutralization assay by plasma from all four vaccinees, but there was no plasma tier 2 neutralizing activity seen (S3B Fig).

**Fig 1:**
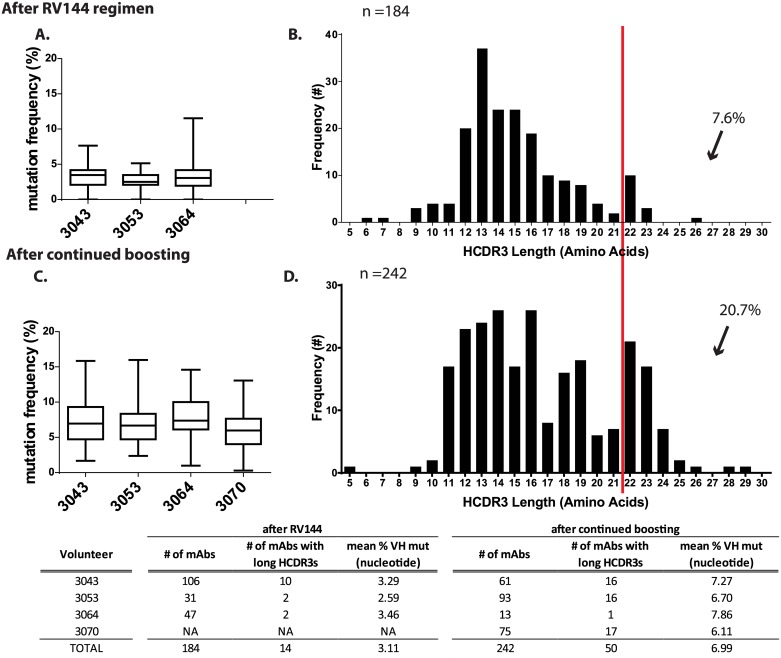
Boosting of RV144 vaccinees increased V_H_ chain gene mutation frequency and caused a repertoire shift, increasing the frequency of antibodies with Heavy Chain Complementary Determinant Region 3 (HCDR3) ≥ 22 amino acids. PBMCs from four vaccinees post-RV144 (A&B) and post-RV305 (C&D) were antigen-specific single-cell sorted with fluorophore labeled conjugates. The V_H_/V_L_ chain genes were PCR-amplified and screened for Env-reactivity by ELISA. The V_H_ chain gene mutation frequency and HCDR3 lengths of 145 Env-reactive antibodies from RV135/144 and 242 Env-reactive antibodies from RV305 were analyzed with Cloanalyst[56]. (A&C) Error bars represent the standard error of the mean.

We isolated AE.A244 gp120 Env-specific post-RV305 boost memory B cells from the four vaccinees- 3043, 3070, 3064 and 3053 (S4 Fig) and from the same vaccinees post-RV144 samples for three of the four vaccinees for whom PBMCs were available. Comparison of the gp120-reactive mAbs from post-RV144 (n = 184 mAbs) with the gp120-reactive mAbs from post-RV305 (n = 242 mAbs) showed that the mean V_H_ nucleotide mutation frequency increased over 2-fold in each vaccinee from a mean of 3.1% to 6.9% (Fig 1A and 1C).

Mobilizing and expanding the pool of long HCDR3 antibodies will be critical for the eventual induction of V2-glycan, V3-glycan, or HCDR3-loop binding bnAbs since many of these bnAbs have HCDR3s longer than 22 amino acids (aa) [24–28]. A meta-analysis of antibodies isolated from post-RV144 studies found that the frequency of Env-reactive B cells with HCDR3s ≥ 22 aa was 2.1%. An analysis of the post-RV305 antibodies indicated that the frequency of Env-reactive B cells with HCDR3s ≥ 22 aa was 20.7% (S5 Fig). To confirm that the increased frequency of Env-reactive long HCDR3 mAbs was related to late boosting, we analyzed the B cell repertoires of three of the four vaccinees (3043, 3053 and 3064) for whom blood samples were available both 2 weeks after the initial RV144 immunization and 2 weeks after the RV305 immunizations. The average frequency of Env-reactive long HCDR3 antibodies within the same vaccinees increased from 7.6% to 20.7%. (Fig 1B and 1D). The HCDR3 length is dictated primarily by V(D)J recombination and can be diversified through secondary means: VH replacement, D-D fusion, insertions, N-nucleotide addition and P-nucelotide addition. Long HCDR3 antibodies have been shown to be biased towards D_H_2, D_H_3 gene and J_H_6 gene segment usage [29]. Coinciding with this observation 72% of the Env-reactive long HCDR3 antibodies isolated post-RV305 and utilized D_H_2 or D_H_3 and 58% used J_H_6 (S1 Table). To determine if this phenomenon was unique to B cell repertoires from late boosting of RV144 vaccinees, we compared these data with the frequency of Env-reactive long HCDR3 found in other HIV-1 Env based immunization regimens. In the GSK PRO HIV-002 human clinical trial, vaccine-recipients received gp120 immunizations in AS01B adjuvant, and the frequency of Env-reactive mAbs with long HCDR3s was 6.9% (n = 58) [30]. In the DNA prime Ad5 boost HIV-1 vaccine regimen used in the HVTN 505 efficacy trial, the frequency of gp140-reactive mAbs with long HCDR3s was 4.1% [31] (S2 Table p< 0.05 compared to RV305 boost data; Fisher’s Exact Test). These data suggested that other immunization regimens without boosting did not expand memory B cell pools with long HCDR3s to the extent achieved with the RV305 boosts.

In vaccinee 3053, seven gp120-reactive B cell clonal lineages were present after the initial RV144 vaccine regimen that persisted and had expanded after boosting 6–8 years later in RV305, one of which, DH678, had a long HCDR3. In vaccinee 3043 nine gp120-reactive B cell clonal lineages were identified after RV144 that were also represented in the samples taken after the RV305 boosts. The antibodies in two of these lineages, DH686 and DH576, had long HCDR3s (S6 Fig). These data demonstrate that memory B cells producing antibodies with long HCDR3s were induced by the initial RV144 regimen and could be expanded with boosting 6–8 years later.

### Epitope mapping of gp120-reactive long HCDR3 antibodies

**Fig 2:**
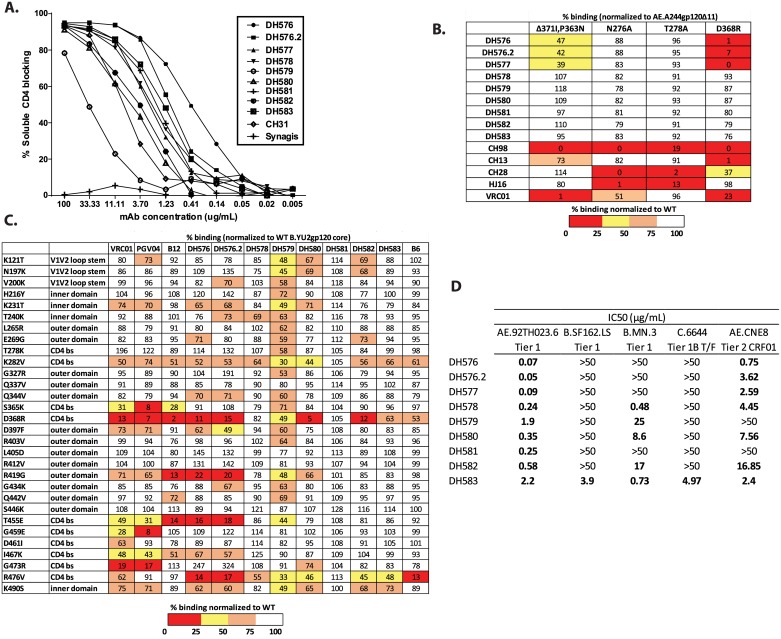
The long Heavy Chain Determinant Region 3 (HCDR3) antibodies that neutralize virus bind the CD4 binding site (CD4 bs). Purified recombinant monoclonal antibodies (mAbs) were assayed by ELISA for (A) blocking the binding of soluble CD4 to AE.A244gp120, and (B) sensitivity to the CD4 binding site mutations Δ371I/P363N, D368R, N276A, and T278A in the AE.A244gp120 protein. (C) Epitope mapping of mAbs on yeast displayed YU2gp120 with point mutations within the inner domain, outer domain and CD4 binding site. (D) Assaying the long HCDR3 CD4 bs antibodies for HIV-1 neutralization in the TZM-bl neutralization assay.

All antibodies isolated were assayed by ELISA as transient transfection supernatants and we selected twenty-seven Env-binding antibodies derived from blood memory B cells post-RV305 boosts based on HCDR3-length (≥ 22 aa) as a representative set of antibodies for characterization (S3 Table). Nine of the 27 mAbs neutralized the neutralization sensitive (tier-1) virus AE.92TH023 in the TZM-bl neutralization assay [23,32,33](S4 Table). The epitopes of these nine neutralizing mAbs with long HCDR3s were then mapped by ELISA for activity in blocking soluble (s) CD4 binding to Env and for binding to mutant Envs. All 9 long-HCDR3 antibodies that neutralized HIV-1 blocked sCD4 binding by ≥70% (Fig 2A) and also blocked binding of CD4 bs bnAbs VRC01 and CH31 (S7 Fig).

Env mutations I371, P363, R476 and D368 generally reduce binding by CD4bs Abs [34]. When assayed with Δ371I/P363N and D368R CD4bs Env mutants, binding of three neutralizing mAbs (DH576, DH576.2, and DH577) was measurably lower compared to wild-type Env (Fig 2B). Seven of nine long HCDR3 sCD4 blocking mAbs (Fig 2A) bound to B.YU2gp120. The binding epitopes of these seven mAbs were mapped by yeast display using B.YU2gp120 core (ΔV1, V2, V3 loops) and B.YU2gp120 cores with mutations that reduce binding by known CD4bs Abs [35]. In contrast to epitope mapping on A244gp120 Env binding of six of seven mAbs were D368R sensitive (Fig 2C). The four Abs not sensitive to the D368R mutation in A244gp120 likely have a higher affinity for the A244gp120 protein then YU2gp120 and their epitope is less dependent on Env D368. Abs DH576 and DH576.2 shared with the CD4bs bnAb B12 sensitivity to 3 CD4bs-critical mutations (D368R, R419G, T455E) and 2 of 3 additional mutation sensitivities (K282V and I467K) [36,37] suggesting these vaccine-induced CD4bs mAbs have a specificity more similar to that of B12 than to that of the non-bnAb CD4bs mAb, B6 which is not sensitive to D368R, R419G and T455E mutations (Fig 2C).

In the TZM-bl cell assay, all neutralizing CD4bs mAbs neutralized not only AE.92TH023 but also the heterologous tier 2 CRF01 isolate AE.CNE8 isolate. DH583 was the broadest neutralizing antibody, also neutralizing the tier 1 viruses B.SF162, B.MN, and the tier 1B (intermediate neutralization sensitivity) primary isolate C.6644 (Fig 2D). Long HCDR3 neutralizing mAbs were assayed against four additional tier 2 CRF01 AE isolates but showed no additional neutralization breadth (S5 Table).

In RV144, infection risk correlated inversely with V1V2 antibody responses [8]. Two V1V2 binding antibodies, CH58 and CH59, neutralized the autologous tier 1 isolate AE.92TH023 in the TZM-bl neutralization assay and also mediated ADCC against tier 2 virus infected cells [9]. To determine whether the long HCDR3 CD4bs mAbs isolated after the RV305 boosts also mediated ADCC, the Abs were expressed in an IgG1 backbone optimized for FcγRIIIa binding[38] and assayed for ADCC against virus-infected cells. DH583 mediated ADCC against B.WITO and C.1086C virus infected cells, with an endpoint concentration of approximately 0.1μg/ml and overall ADCC activity, as evaluated by positive area under the dilution curve, similar to that observed for the CD4bs bnAb CH31. The other eight long HCDR3 CD4 bs mAbs had little to no ADCC activity against any of the isolates tested (S8 Fig).

### The germline precursors of vaccine-induced long HCDR3 CD4bs antibodies neutralize the tier 2 HIV-1 strain, CRF01 AE.CNE8

**Fig 3:**
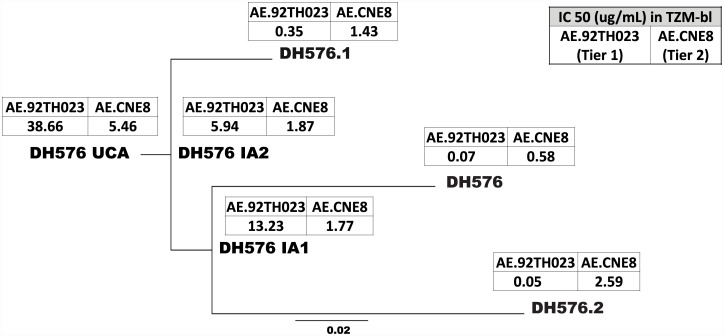
Neutralization of the tier 1 HIV-1 isolate AE.92TH023 and the tier 2 HIV-1 isolate CRF_01 AE.CNE8 by the DH576 Unmutated Common Ancestor (UCA) and DH576 lineage members. The DH576 clonal lineage was inferred with Cloanalyst[56]. The DH576 clonal lineage was assayed for neutralization of the tier 1 isolate AE.92TH023 and the tier 2 isolate CRF_01 AE.CNE8 in the TZM-bl neutralization assay. Affinity maturation of the DH576 clonal lineage improved neutralization for the tier 1 isolate AE.92TH023 by > 3 logs and for the tier 2 isolate CRF_01 AE.CNE8 by approximately 1 log.

The most heavily mutated member of the long HCDR3 CD4bs DH576 B cell clonal lineage was DH576.2 (V_H_ nucleotide mutations of 10.33%), but the additional mutations did not broaden or strengthen HIV-1 tier 2 CRF01 AE.CNE8 neutralization (Fig 2) with respect to neutralization by less mutated lineage members such as DH576 (V_H_ mutations of 7.33%) (S3 Table). To determine the effects of affinity maturation, we assayed the UCA, IAs and three naturally occurring DH576 clonal lineage mAbs for neutralization of the autologous tier 1 virus AE.92TH023 and the heterologous tier 2 virus CRF01 AE.CNE8. The DH576 UCA neutralized both the tier 1 HIV-1 AE.92TH023 and the tier 2 HIV-1 CRF01 AE.CNE8. As affinity maturation progressed, there was a difference in the ratio of neutralization potencies for tier 1 and tier 2 viruses. Affinity maturation increased DH576 ineage neutralization potency (IC_50_) against the tier 1 AE.92TH023 by over 3 logs, but increased its potency (IC_50_) against the tier 2 AE.CNE8 by less than 1 log (Fig 3). These data can be explained in part as follows. The UCA of DH576 had a higher affinity for AE.CNE8gp120 than did the UCA for AE.A244gp120 (nearly identical in sequence to AE.92TH023). Binding assays to the two gp120s showed affinity maturation of < 1 log to AE.CNE8gp120 while there was > 2 log increase in affinity maturartion for AE.A244gp120 (S6 Table).

To determine whether neutralization of HIV by a UCA was a common property of HCDR3-loop CD4 bs binding mAbs, we assayed the UCAs of the other vaccine-induced CD4bs mAbs and found that 3 of 8 nAb UCAs neutralized both AE.92TH023 and AE.CNE8 (S7 Table).These data indicated that the vaccination regimens in both RV144 and RV305 trials could elicit long HCDR3 CD4bs mAbs, whose germline genes could mediate tier 2 neutralization of HIV-1 AE.CNE8.

### Structural analysis of long HCDR3 vaccine-induced CD4bs antibodies

**Fig 4:**
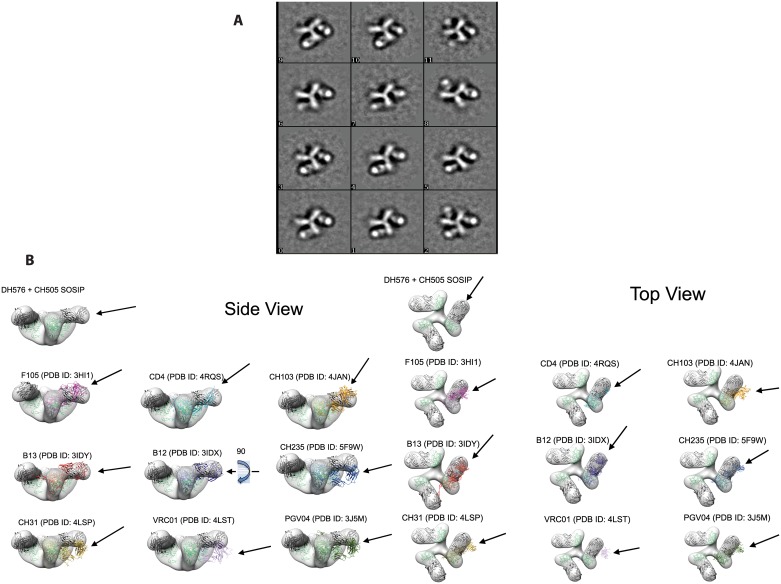
Negative stained electron microscopy of DH576 in complex with CH505 SOSIP.664. (A) 2D class averages of one, two, or three DH576 Fabs bound to the trimer and (B) Side and top views of the 3D EM reconstruction with the indicated Ab or sCD4 superimposed on the gp120 subunit for comparison with DH576 binding. Arrows indicate the angle at which the indicated antibody approaches the trimer.

Progression from sporadic tier 2 neutralization to increased tier 2 virus neutralization breadth depends upon the epitope specificity [39] and the precise footprint of the Ab on Env [26]. We analyzed by negative stain electron microscopy (EM) a CH505 SOSIP.664 trimer bound with DH576. A 3D reconstruction showed DH576 bound to an open trimer—that is, to Env in a conformation related to the one stabilized by CD4 binding (Fig 4A, S9 Fig). A top view of the complex suggested that the DH576 footprint might resemble those of bnAbs B12 and CH103 (Fig 4B). The bnAbs CH103, CH235, CH31, VRC01, and PGV04, as well as CD4 itself, project away from the center of the trimer, avoiding interference with adjacent gp120 subunits in the closed trimer conformation, whereas DH576 may require the open form in order to avoid overlap. The DH576 Fab has an orientation with respect to Env quite similar to that of the B12 Fab, but turned by ~90° about its long axis (Fig 4B).

The CD4bs bnAb B12 interaction with gp120 depends upon an aromatic residue at the apex of the HCDR3 loop, aromatic residues around the base of the HCDR3 region, a tyrosine at the apex of the HCDR2 loop and positively charged amino acids in the LCDR1[40]. An alignment of the DH576 inferred UCA and naturally occurring clonal lineage members with the B12 heavy sequence showed that, like B12, the DH576 clonal lineage contained an aromatic residue at the apex of the HCDR3 loop, aromatic residues around the base of the HCDR3 and a tyrosine in the HCDR2 loop (S10 Fig). The HCDR3s of B12 and DH576 protrude at different angles and when DH576 is superimposed on the B12-gp120 complex, the HCDR3 of DH576 sterically clashes with gp120. Thus it is not suprising that DH576 rotates by approximately ~90° when it binds to gp120 (S10 Fig).

Negative stain EM of 92Br SOSIP.664 with DH583, the broadest mAb identified, showed that DH583 also binds an open form of the trimer, even though this trimer is stable in the closed form (S11 Fig). These observations suggest that antibodies elicited in the RV305 trial bind epitopes generally shielded in closed trimers, consistent with the use of gp120 (rather than a closed Env trimer) as a principal component of the original, RV144 vaccine.

## Discussion

In this paper we demonstrate that late (6–8 year) boosting of RV144 vaccinees with ALVAC-HIV and AIDSVAX gp120 B/E increased the V_H_ chain gene mutation frequency and expanded clonal lineages of CD4bs antibodies with long HCDR3 regions. Increased somatic hypermutation and affinity maturation by repetitive immunization with a gp120-protein has previously been reported in humans and non-human primates [30,41]. In this study the boosting of RV144 vaccinees occurred several years later suggesting that in spite of the rapid waning in plasma IgG seen in the RV144 vaccine trial, long lived memory B cells were induced that could be recalled with subsequent boosting. The observation that three CD4bs clonal lineage UCAs could neutralize tier 2 CRF01 AE AE.CNE8 raised the hypothesis that the AE.A244 gp120 Env in the boost selectively stimulated expansion of a pool of pre-existing tier 2 neutralizing clonal lineages. An antibody HCDR3 arises from recombination of immunoglobulin heavy variable (V_H_), diversity (D_H_), and joining (J_H_) genes; its overall length is determined by gene usage [20,29,42], D-D fusion [25,42,43], N nucleotide additions [22,42], or V_H_ gene replacement [44,45]. While B cells that give rise to long HCDR3 antibodies frequently undergo productive gene rearrangement [42], they can experience negative selection during B cell development because of autoreactivity or polyreactivity [21,22]. Thus, in uninfected individuals, only approximately 4% of the naïve repertoire consists of long HCDR3 antibodies, and this population contracts by ~ 50% due to negative selection in the bone marrow at the first immune tolerance checkpoint [22,29].

Virus neutralization by a fully reverted, inferred UCA has been reported for V1V2 and CD4bs bnAbs [25,46–49] that came from HIV-1 chronically infected individuals. Pancera et al [49] and Bonsignori et al [25] found that V1V2 bnAb UCAs of PG16 and CH01 could neutralize several primary HIV strains. Both UCAs neutralized clade C ZM233, clade A AQ23 and clade B WITO[25,49]. More recently Gorman et al [47] and Andrabi et al [48] have shown that the combining sites of multiple V1V2 bnAbs share binding motifs, and their UCAs frequently neutralize the same HIV-1 primary isolates, suggesting that these primary isolate Envs might be candidates for use as immunogens.

The fundamental question raised is whether the CD4bs B cell clonal lineages primed by RV144 and expanded with the repetitive boost of the same vaccine can, with continued boosting, affinity mature into bnAbs. The epitopes of the vaccine-induced CD4bs mAbs described here appear to overlap those of other CD4bs antibodies and that of bnAb B12 in particular. Electron microscopy of negatively stained complexes showed that the vaccine-induced mAbs DH576 and DH583 bound an open form of Env, consistent with a gp120 being used in the vaccine-regimen, and the images were consistent with the CD4bs epitope mapping. Seven of the nine long HCDR3 CD4bs mAbs characterized here had the same V_H_3 gene usage as the CD4 bs bnAbs CH98 [36] and HJ16[50]; one of the nine used V_H_1-69 (S3 Table), like VRC13 [26]. One mAb also used a V_L_ κ4–1 like HJ16 and six of the nine long HCDR3 CD4 bs mAbs used either a V_L_ κ3–20 or V_L_ κ1–33, which are V_L_ chain genes used by the CD4bs bnAbs B12, VRC01, VRC-PGV04, VRC30-34, 3BNC117, 3BNC60, NIH45-46, 12A12, 12A21 and 8ANC131 (reviewed in [16]). Nonethless, after 6–8 years and 4 boosts, the induced mAbs neutralized only 1 of 40 tier 2 viruses that were assayed with DH583 and DH576. Moreover, the neutralizing IC_50_ of the DH576 clonal lineage for CRF01 AE.CNE8 changed only marginally during affinity maturation, strongly suggesting that theAE.A244gp120, although it could bind to the UCA, did not select clonal lineage members that could undergo affinity maturation and exhibit greater breadth. Rather it was only neutralization of the tier 1 virus AE.92TH023 for which vaccine boosting led to a 3 log increase in IC_50_. Thus, it is likely that AE.A244 gp120 selected antibody responses that neutralized viruses with an “open” Env conformation, consistent with known conformational properties of the free gp120 fragment. As previously shown in non-human primates antibodies that exclusively bind an open Env sterically clash with Env variable regions leaving little chance of maturing to a bnAb [51,52]. We do not yet know whether a *de novo* series of prime-boost immunizations with stable, closed trimer as proposed by others [51,53,54] would engage the UCAs of long HCDR3 antibodies such as DH576 and induce affinity maturation to neutralization breadth. In general, Envs of tier 1 viruses open readily, while those of tier 2 viruses do not. The Env of CRF01 AE.CNE8 apparently opens readily enough to bind the antibodies we have characterized, but most other tier 2 Envs do not. The boosts that expanded the pool of long HCDR3 mAbs occurred several years after the completion of the RV144 trial. We do not know what effect the interval between boosting has on the vaccine-induced antibody repertoire. In the RV306 HIV-1 clinical trial (NCT01931358), vaccine-recipients received the same ALVAC-HIV and AIDSVAX B/E prime-boost regimen and were boosted again with a shorter rest period. Characterization of the Env-reactive mAb repertoire in these vaccine-recipients may provide some insight into whether the length of the rest period necessary for expansion of long HCDR3 mAbs.

In summary, study of the B cell repertoires of memory B cells induced by the RV305 trial vaccine-regimen has defined a set of CD4bs-reactive B cell clonal lineages that were initiated by the RV144 vaccine-regimen and expanded after late boosting with the ALVAC-HIV and AIDSVAX B/E immunogens. These antibodies derived from UCAs with some degree of tier 2 virus neutralization capability.

## Materials and methods

### Ethics statement

The RV305 clinical trial (NCT01435135) received approvals from Walter Reed Army Institute of Research, Thai Ministry of Public Health, Royal Thai Army Medical Department, Faculty of Tropical Medicine, Mahidol University, Chulalongkorn University Faculty of Medicine, and Siriraj Hospital. Written informed consent was obtained from all clinical trial participants.The Duke University Health System Institutional Review Board approved all human specimen handling.

### Donor subjects

The RV305 clinical trial (NCT01435135) was a randomized double blinded placebo-controlled boosting of 162 RV144 clinical trial participants (NCT00223080) that occurred in Thailand. The RV305 clinical trial was sponsored by the U.S. Army Office of the Surgeon General and conducted in collaboration with the U.S. Army Medical Research and Materiel Command and the Thailand Ministry of Public Health. The primary objective was to characterize the cellular and humoral immune response after boosting and to evaluate the safety and tolerability of late and repetitive boosting with the ALVAC-HIV (vCP1521) and AIDSVAX B/E immunogens. Six-eight years after the conclusion of RV144, RV305 volunteers were randomized into three groups and boosted two times with a six month interval with either AIDSVAX B/E + ALVAV-HIV (vCP1521), AIDSVAX B/E or ALVAC-HIV (vCP1521) or a placebo. After commencement no changes were made to the vaccine-regimen. All HIV-1 uninfected RV144 participants that had completed the full RV144 vaccine-regimen, were at low risk for HIV-1 infection based on self-reported behavioral habits, able to pass a Test of Understanding, gave written consent and were in general good health were eligible. Female volunteers had to be on adequate birth control 45 days prior to the first inject and consent to remaining on birth control. For safety reasons women that were pregnant, nursing or planning on becoming pregnant were excluded. Volunteers with a conflict of interest, psychological or medical conditions, or those unable to complete a Test of Understanding were excluded. Vaccine safety was measured by self-reporting on a diary card local and systemic reactions for three days post-vaccination. All adverse events and serious adverse events were recorded throughout the trial and up to three months post final boost.

### Antigen-specific single-cell sorting

Peripheral blood mononuclear cells (PBMCs) were stained with Aqua vital dye ((AqVd) Invitrogen), IgM-FITC, IgD-PE, CD3 -PECy5, CD14-BV605, CD16-BV570, CD235a-PECy5, CD27-PECy7, CD38-APC-AF700, CD19-APCCy7, along with AF647 and BV421 conjugated antigens. Viable antigen-specific B cells (AqVd-CD14-CD16-CD3-CD235a-CD19+IgD-CD38all, AF647 and BV421 double positive) were single-cell sorted with a BD FACSAria II- SORP (BD Biosciences, Mountain View, CA) into 96 well PCR plates and stored at -80°C.

### Single-cell PCR and sequencing

Immunoglobulin variable heavy and light chain variable regions (V_H_ and V_L_) were RT-PCR amplified using AmpliTaq360 Master Mix (Applied Biosystems) with conditions previously described [55]. PCR products were purified (Qiagen, Valencia, CA) and sequenced with a BigDye Sequencing kit (Applied Biosystems) on an ABI 3700 sequencer. V_H_ and V_L_ chain gene rearrangements, clonal relatedness, UCA and intermediate ancestor (IA) inferences were made using Cloanalyst [56].

### Monoclonal antibody production

PCR-amplifed sequences were transiently expressed as previously described [55]. Briefly, linear expression cassettes were constructed by placing the PCR-amplified V_H_ and V_L_ chain genes under the control of a CMV promoter along with an IgG constant region and poly A signal sequence. These linear expression cassettes were then co-transfected into 293T cells and after three days the cell culture supernatants were harvested and concentrated.

For large scale expression, the V_H_D_H_J_H_ and V_L_J_L_ genes were synthesized (V_H_ chain in the IgG1 4A backbone) and transformed into DH5α cells (GeneScript, Piscataway, NJ). Plasmids were expressed in Luria Broth, purified (Qiagen, Valencia, CA) and ~ 5x10^6^ 293i cells were transfected with 1 mg of Ig (V_H_) and light (V_L_) chain genes using poly-ethylenimine (PEI) or with 0.4mgs of heavy- and light chain-gene using ExpiFectamine^™^ (Life Technologies, Carlsbad, CA) following the manufacturers protocol. After five days mAbs were concentrated, purified from the cell culture supernatant by an overnight incubation with Protein A beads and buffer exchanged into PBS.

### Plasma and mAb binding assays

High affinity 384-well microplates (Costar 3700) were coated overnight at 4°C with 30ng/well of protein in 0.1% Sodium Biocarbonate. For binding, a direct ELISA was performed in which monoclonal antibodies (mAbs) beginning at 100ug/mL were diluted 3-fold in blocking buffer and added to the plates for 1 hour. Antibody binding was detected using IgG-HRP (Rockland, Limerick, PA) diluted 1:10,000 in azide-free blocking buffer. For the blocking ELISA, mAbs of interest were diluted and added to the plate for one hour. Plates were washed and a biotinylated mAb was added for one hour. Blocking was evaluated by adding streptavidin-HRP. The direct binding and blocking ELISAs were developed using SureBlue Reserve TMB One Component microwell peroxidase substrate (catalog no. 53-00-03; KPL) and the reactions were stopped with 0.1% HCL. Plates were read on a plate reader (Molecular Devices) at 450 nm. Palivizumab (Synagis) (MedImmune, LLC; Gaithersburg, MD) was used as a negative control. The plasma was screened with the binding Ab multiplex assay (BAMA) as previously described. The antibody B12 was a gift from QBI and the Vaccine Research Program, Division of AIDS, NIAID contract # HSN272201100023C.

### Neutralization assays

Neutralization assays were performed in both TZM-bl and A3R5 cell lines as previously described [23,32]. Data were reported as ID_50_ titers for plasma and IC_50_ titers for mAbs.

### Yeast display epitope mapping of mAbs

Purified mAbs were epitope mapped on B.YU2gp120 core proteins (ΔV1, V2, V3 loops) displayed on *S*. *cerevisiae* as previously described [35,36]. Briefly, mAbs that bound B.YU2gp120 core protein were assayed for binding to 31 different B.YU2gp120 core proteins with point-mutations and the wild type protein. Antigen-specific recognition was confirmed by the observation that mAbs did not show binding to non-displaying *S*. *cerevisiae*. Data was recorded as the percent binding to a mutant relative to the wild type core proteins. The B12 binding data in Figure 3 are from [36].

### Surface plasmon resonance

Surface plasmon resonance was performed on a BIAcore 4000 instrument. The purified recombinant mAb was immobilized to a CM5 sensor chip and envelope binding was measured in real time with continuous flow of PBS (150mM NaCL, 0.005% surfactant P20 [pH 7.4] at 10–30 μl/min. Data was analyzed with BIAevaluation 4.1 software (BIAcore).

### ADCC assays

ADCC mediated by the mAbs was assessed according to previously published procedures [57,58]. Briefly, HIV-1 reporter virus used was a replication-competent infectious molecular clone (IMC) designed to encode the HIV-1 *env* genes in *cis* within an isogenic backbone that also expresses the *Renilla* luciferase reporter gene and preserves all viral open reading frames [59]. CEM.NKR_CCR5_ cells (NIH AIDS Reagent Program, Division of AIDS, NIAID, NIH: CEM.NKR CCR5+ Cells from Dr. Alexandra Trkola [60] were infected with HIV-1 IMCs encoding the subtype AE CM235 (accession number AF259954), B WITO (accession number JN944948), and C Ce1086.c (accession number FJ444395) *env* genes within an NL4-3 backbone [59]. Whole PBMC from an HIV-seronegative donor with the heterozygous 158F/V genotype for Fc-gamma receptor IIIa were used as effector cells at an effector cell to target cell (E:T) ratio of 30:1. MAb A32 (James Robinson; Tulane University, New Orleans, LA), Palivizumab (MedImmune, LLC; Gaithersburg, MD; used as negative control) and vaccine induced mAbs were tested at a final concentration range of 10–0.039μg/ml using 4-fold serial dilutions. All the conditions were evaluated after 6 hour incubation at 37°C and 5%CO_2_. The ADCC activity was reported as % specific killing calculated as [(RLU in control well − RLU in test well)/ RLU of control well] ×100. The results were considered positive if ADCC activity was ≥15% specific killing. ADCC activities are reported either as the endpoint concentration (EC), defined as the mAb concentration that intersects the positive cutoff of 15% specific killing, or as positive area under the curve (pAUC), calculated by the trapezoidal rule using the values ≥15% specific killing.

### Design of the CH505 SOSIP.664 construct

To generate the autologous HIV-1 CH505 SOSIP.664 and clade B 92Br SOSIP.664 expression constructs we followed established SOSIP design parameters [61]. Briefly, the SOSIP.664 trimer was engineered with a disulfide linkage between gp120 and gp41 by introducing A501C and T605C mutations (HxB2 numbering system) that covalently links the two subunits of the heterodimer [61]. The I559P mutation was included in the heptad repeat region 1 (HR1) of gp41 for trimer stabilization, and a deletion of part of the hydrophobic membrane proximal external region (MPER), in this case residues 664–681 of the Env ectodomain [61]. The furin cleavage site between gp120 and gp41 (_508_REKR_511_) was altered to _506_RRRRRR_511_ to enhance cleavage [61]. The resulting, codon-optimized CH505 SOSIP.664 *env* gene was obtained from GenScript (Piscataway, NJ) and cloned into pVRC-8400 using Nhe1 and NotI restriction sites and the tissue plasminogen activator signal sequence.

### Expression and purification of Fabs for structural analysis

Fabs were expressed by transient transfection of HEK 293F suspension cells, using linear PEI following the manufacturer’s suggested protocol. After 5 d, supernatants were clarified by centrifugation and diluted twofold with 1x PBS buffer, and the protein isolated from the diluted spernatant using CaptureSelect LC-Kappa (Hu) affinity matrix (Thermo Fisher Scientific, Waltham, MA), according to manufacturer’s protocols. Fractions containing the protein of interest were pooled, concentrated, and further purified by gel filtration chromatography using a Superdex 200 analytical column (GE Healthcare Life Sciences, Pittsburgh, PA) in a buffer of 2.5mM Tris, pH 7.5, 350mM NaCl, and 0.02% sodium azide.

### Purification of Envs for analysis by negative stain EM

Each SOSIP.664 construct was transfected into 293F cells together with a plasmid encoding the cellular protease, furin, at a 4:1 Env:furin ratio. The cells were allowed to express the soluble trimer for 5–7 days. Culture supernatants were collected, cells removed by centrifugation at 3800 x g for 20 min, and the supernatant filtered with a 0.2 μm pore size filter. The soluble SOSIP was purified by flowing the filtered supernatant over a lectin (*Galanthus nivalis*) affinity chromatography column overnight at 4°C. The lectin column was washed with 1x PBS, followed with 1x PBS supplemented with 0.5 M NaCl, and proteins were eluted with 1 M methyl-α-D-mannopyranoside dissolved in 1x PBS. The eluate was concentrated and loaded for further purification onto a Superdex 200 10/300 GL column (GE Healthcare Life Sciences, Pittsburgh, PA) prequilibrated in a buffer of 5 mM Hepes, pH 7.5, 150 mM NaCl and 0.02% sodium azide for analysis by EM.

### Electron microscopy

Purified SOSIP.664 trimer was incubated with a five molar excess of Fab at 4°C for 1 hour. A 3 μL aliquot containing ~0.01 mg/ml of the complex was applied for 30 s onto a carbon coated 400 Cu mesh grid that had been glow discharged at 20 mA for 30 s, followed by negative staining with 2% uranyl formate for 20 s. Samples were imaged using a FEI Tecnai T12 microscope operating at 120kV, at a magnification of 52,000x, resulting in a pixel size of 2.13 Å at the specimen plane. Images were acquired with a Gatan 2K CCD camera using a nominal defocus of 1500 nm at 10° tilt increments, up to 50°. The tilts provided additional particle orientations to improve the image reconstructions.

### Negative stain image processing and 3D reconstruction

Particles were picked semi-automatically using EMAN2 [62] and put into a particle stack. Initial, reference-free, two-dimensional (2D) class averages were calculated and particles corresponding to complexes (with one, two, or three Fabs bound) were selected into a substack for determination of an initial model for the DH576: CH505 SOSIP.664 complex. The initial model was calculated in EMAN2, imposing 3-fold symmetry, and subsequent refinement in EMAN2 also imposed 3-fold symmetry. In total, 22,929 particles were included in the final reconstruction. The resolution of the final model was determined using a Fourier Shell Correlation (FSC) cut-off of 0.5.

### Model fitting into the EM reconstructions

The cryo-ET structure of b12-bound gp120 trimer (PDB ID: 3DNL) [63] and an Fab model were manually docked into the EM density and refined with the UCSF Chimera ‘Fit in map’ function [64]. The gp120 subunit of crystal structures with different Fabs were superposed on other gp120 cores from the PDB by least-squares fitting in Coot [65]

### Crystallization, structure determination, and refinement

The DH576 Fab was crystallized at 10–15 mg/mL. Crystals were grown in 96-well format using hanging drop vapor diffusion and appeared after 24–48 h at 20°C. Fab crystals were obtained in the following conditions: 20% PEG 4000, 100mM Hepes, pH 7.0, 1M NaCl. Crystals were harvested and cryoprotected by the addition of 20–25% glycerol to the reservoir solution and then flash-cooled in liquid nitrogen.

Diffraction data were obtained at 100 K from beam line 24-ID-C at the Advanced Photon Source using a single wavelength. Datasets from individual crystals were processed with HKL2000[66]. Molecular replacement calculations for the free Fab were carried out with PHASER[67], using the variable domains of PGT135 [Protein Data Bank (PDB) ID 4JM2] and the constant domains of VRC01 from the VRC01/gp120 complex [Protein Data Bank (PDB) ID 4LSS] as the starting models for molecular replacement.

Refinement was carried out with PHENIX[68], and all model modifications were carried out with Coot[65]. During refinement, maps were generated from combinations of positional, group B-factor, and TLS (translation/libration/screw) refinement algorithms. Secondary-structure restraints were included at all stages for all Fabs. Structure validations were performed periodically during refinement using the MolProbity server[69]. The final refinement statistics are summarized in (S8 Table).

### Statistical analysis

All statistical analysis was performed in SAS by the Duke Human Vaccine Institute statistical team. The statistical test and p value are recorded where used.

### Accession numbers

The EM reconstruction has been deposited in the Electron Microscopy Data Bank as EMD-8573. The crystal structure of DH576 has been deposited in the Protein Data Bank as PDB ID5UIX. The V_H_ and V_L_ chain genes described have been submitted to Genbank with accessioning numbers KY499910-KY499949.

## Supporting information

S1 Fig The RV144 and RV305 immunization regimens. The RV305 clinical trial (NCT01435135) was a placebo-controlled double-blinded HIV-1 vaccine trial that occurred six-eight years after the conclusion of RV144 (NCT00223080). RV144 vaccinees that received the full RV144 immunization regimen and were HIV-1 seronegative were split into three groups and boosted two times with either ALVAC-HIV + AIDSVAX B/E (Group I), AIDSVAX B/E (Group II), or ALVAC-HIV alone (Group III).
(EPS)

S2 Fig Plasma neutralization of RV305 vaccinees. The plasma from two weeks after the second boost in RV305 (wk. 26) from vaccinees boosted with AIDSVAX B/E (Group I), AIDSVAX B/E + ALVAC-HIV (Group II), or ALVAC-HIV (Group III) were screened in the A3R5 neutralization assay for neutralization of a panel of HIV-1 CRF_01 AE isolates. (A) ID50 in A3R5 cells of each CRF_01 isolate per group (Grp I and III n = 23 and Grp II n = 24). (B) Individual ID50 in A3R5 neutralization assay of vaccines from Group I and Group II when assayed against the 11 CRF_01 AE viruses in (A). Red arrows denote vaccinees selected for antibody isolation.
(EPS)

S3 Fig Plasma gp120-specific IgG binding and neutralization of vaccine-recipients whose vaccine-induced memory B cell repertoires were analyzed. The plasma of the four vaccinees selected for antibody isolation were longitudinally assayed for (A) IgG binding to the autologous isolate AE.A244gp120Δ11 and heterologous isolate B.6240gp120Δ11 by Luminex and (B) plasma neutralization of tier 1 and tier 2 viruses in the TZM-bl neutralization assay.
(EPS)

S4 Fig The isolation of AE.A244gp120-specific antibodies from four RV305 vaccinees. AE.A244gp120-double positive (upper right quadrant)memory B cells from two weeks after the second RV305 boost (wk. 26) were antigen-specific single-cell sorted with fluorophore labeled conjugates for RT-PCR.
(EPS)

S5 Fig Relative to RV144 the gp120-specific antibodies after continued boosting had a greater frequency of antibodies with Heavy Chain Complementary Determinant Region 3 (HCDR3) ≥ 22 amino acids. PBMCs from (A)14 RV135/144 vaccinees and (B) 4 RV305 vaccinees were antigen-specific single-cell sorted with fluorophore labeled conjugates. The V_H_/V_L_ chain genes were PCR-amplified and screened for Env-reactivity by ELISA. The V_H_ chain gene mutation frequency and HCDR3 lengths of 145 Env-reactive antibodies from RV135/144 and 242 Env-reactive antibodies from RV305 were analyzed with Cloanalyst[56].
(EPS)

S6 Fig Env-reactive persistent clonal lineages identified in two vaccinees that were initiated in RV144 vaccine-regimen and boosted 6–8 years later with the RV305 vaccine-regimen. PBMCs from two vaccinees that were immunized in both the RV144 and RV305 vaccine trials were antigen-specific single-cell sorted with fluorophore labeled conjugates. The V_H_/V_L_ chain genes were PCR-amplified and analyzed with Cloanalyst [56].
(EPS)

S7 Fig The long heavy chain determinant region 3 antibodies block the binding of the CD4 bs bnAbs VRC01 and CH31 to AE.A244gp120. Antibodies were diluted 3-fold starting at 100 ug/mL and assayed by ELISA for blocking of the CD4bs bnAbs VRC01 and CH31 binding to AE.A244gp120.
(EPS)

S8 Fig The long Heavy Chain Determinant Region 3 (HCDR3) CD4 binding site (bs) antibodies do not potently mediate Antibody-Dependent Cellular Cytotoxicity (ADCC). The RV305 antibodies were assayed for ADCC against WITO, 1086C and CM235 infected cells. Shown is the antibody (A) end point concentration and (B) area under the curve. For comparison other CD4 bs and the C1/C2 antibody A32 are include.
(EPS)

S9 Fig Negative stain EM of DH576 Fab in complex with the CH505 T/F SOSIP.664 trimer. The Fourier shell correlation curve for the complex is shown along with the resolution determined using FSC = 0.5.
(EPS)

S10 Fig Superposition of B12 (purple) and DH576 (black) Fabs and an alignment of the heavy chain sequences. (A) The HCDRH3 loop is highlighted to indicate the different conformation of this loop for the two Fabs. The panel on the right shows a superposition with the B12-gp120 complex and the arrow points to where the HCDR3 loop of DH576 clashes with gp120 when the two antibodies are superimposed onto gp120 at the same angle. (B) A heavy chain alignment of the DH576 unmutated common ancestor and naturally occurring DH576 clonal lineage members with the CD4 binding site bnAb B12. The * highlights conserved aromatic residues critical for B12 binding [40].
(EPS)

S11 Fig Negative stained electron microscopy of DH583 in complex with 92Br SOSIP.664. 2D class averages of the complex are shown (top two rows) and unliganded trimer in the closed form (bottom row).
(EPS)

S1 Table The long heavy chain determinant region 3 antibodies isolated from post-RV305 by antigen-specific single-cell sorting. PBMCs from four vaccinees post-RV305 were antigen-specific single-cell sorted, PCR-amplified and sequenced. The V_H_/V_L_ chain gene sequences were analyzed with Cloanalyst[56].
(EPS)

S2 Table The boosts to RV144 vaccinees increased the frequency of long HCDR3 mAbs compared to mAbs isolated from other HIV-1 human clinical trials. The frequency of gp120-reactive (RV144 boosts) or gp140-reactive (HVTN 505 and GSK PRO HIV-002) mAbs with HCDR3s ≥ 22 amino acids were compared using the Fisher’s Exact Test. The RV144 boosted vaccinees had a greater frequency of long HCDR3s compared to either the HVTN505 or GSK PRO HIV -002 clinical trials.
(EPS)

S3 Table The long heavy chain determinant region 3 antibodies that were recombinantly expressed in this study. The V_H_/V_L_ chain gene sequences were analyzed with Cloanalyst[56].
(EPS)

S4 Table Assaying the long heavy chain determinant region 3 antibodies for autologous tier 1 neutralization. The antibodies were recombinantly expressed and assayed for neutralization of AE.92TH023 in the TZM-bl neutralization assay.
(EPS)

S5 Table Assaying the long heavy chain determinant region 3 CD4 bs antibodies for neutralization of additional tier 2 CRF_01 AE isolates in the TZM-bl neutralization assay. The antibodies were recombinantly expressed and assayed for neutralization of four tier 2 CRF_01 clade AE isolates in the TZM-bl neutralization assay.
(EPS)

S6 Table Apparent affinity of the DH576 clonal lineage antibodies for clade AE CRF_01 Env binding. The unmutated common ancestor and intermediate antibody sequences were inferred with Cloanalyst[56]. The antibodies were recombinantly expressed and assayed for binding to Envs by surface plasmon resonance. Data shown are the dissociation constant (K_d_).
(EPS)

S7 Table Neutralization of the tier 2 CRF_01 AE.CNE8 virus by the Unmutated Common Ancestor (UCA) of multiple CD4 binding site (bs) clonal lineages. The UCAs of the eight CD4 bs clonal lineages were inferred with Cloanalyst[56]. The antibodies were recombinantly expressed and assayed for neutralization in the TZM-bl neutralization assay.
(EPS)

S8 Table Data collection and refinement statistics. Statistics for highest-resolution shell are shown in parentheses.
(EPS)
